# Do Memory Problems in Older Drivers Impact Driving Frequency in Metro Versus Non-Metro Communities?

**DOI:** 10.1093/geroni/igaa057.509

**Published:** 2020-12-16

**Authors:** Tyler Orr, Randall Rupper

**Affiliations:** 1 Brigham Young University--Provo, Provo, Utah, United States; 2 Salt Lake VA, Salt Lake City, Utah, United States

## Abstract

Perceived memory problems may cause older adults to limit functional activities such as driving. For those individuals living in non-metropolitan communities, greater distances between activities, lack of public transportation, and fewer support systems may make reducing driving frequency less feasible. We hypothesized that older adults in non-metropolitan communities would be more likely to continue frequent driving even if they also perceived memory problems. We used the National Health and Aging Trends Study to examine the association between reported memory difficulty and the frequency of driving. These data were then stratified by metropolitan versus non-metropolitan classification using both ordinal logistic regression and Chi-Squared testing. In both metropolitan and non-metropolitan communities, respondents were more likely to report reductions in driving frequency if they also reported memory problems. However, in both metropolitan and non-metropolitan communities, the majority of respondents reporting fair or poor memory continued to report frequent driving; and, there were no statistical differences in frequency of reported driving between metropolitan and non-metropolitan respondents. These analyses suggest that strategies are necessary in both metropolitan and non-metropolitan areas to help older drivers with perceived memory difficulties to recognize when they need to limit driving. Further research is necessary to determine which strategies are likely to be effective in metropolitan and/or non-metropolitan communities.

